# Early Nasojejunal Nutrition Versus Early Oral Feeding in Patients After Pancreaticoduodenectomy: A Randomized Controlled Trial

**DOI:** 10.3389/fonc.2021.656332

**Published:** 2021-04-29

**Authors:** Xinchun Liu, Qiuyang Chen, Yue Fu, Zipeng Lu, Jianmin Chen, Feng Guo, Qiang Li, Junli Wu, Wentao Gao, Kuirong Jiang, Cuncai Dai, Yi Miao, Jishu Wei

**Affiliations:** ^1^ Pancreas Center, The First Affiliated Hospital of Nanjing Medical University, Nanjing, China; ^2^ Department of General Surgery, Affiliated Hangzhou First People’s Hospital, Zhejiang University School of Medicine, Hangzhou, China

**Keywords:** pancreaticoduodenectomy, delayed gastric emptying, early oral feeding, early nasojejunal nutrition, randomized controlled trial

## Abstract

**Objective:**

The aim of this study was to test the hypothesis that early oral feeding (EOF) is superior to early nasojejunal nutrition (ENN) after pylorus-preserving pancreaticoduodenectomy (PPPD) in terms of delayed gastric emptying (DGE).

**Background:**

DGE is a common complication after PPPD. Although EOF after PPPD is recommended by several international guidelines, there is no randomized trial to support this recommendation.

**Methods:**

From September 2016 to December 2017, a total of 120 patients undergoing PPPD were randomized into the ENN, EOF, or saline groups at a 1:1:1 ratio (40 patients in each group). The primary endpoint was the rate of clinically relevant DGE. Secondary endpoints included overall morbidity, postoperative pancreatic fistula, post-pancreatectomy hemorrhage, abdominal infection, length of hospital stay, reoperation rate, and in-hospital mortality.

**Results:**

The baseline characteristics and operative parameters were comparable between the groups. The incidence of clinically relevant DGE varied significantly among the three groups (ENN, 17.5%; EOF, 10.0%; saline, 32.5%; p =0.038). The saline group had a higher clinically relevant DGE rate than the EOF group (p = 0.014). The saline group also had greater overall morbidities than the ENN and EOF groups (p = 0.041 and p = 0.006, respectively). There were no significant differences in other surgical complication rates or postoperative hospital stay. No mortality was observed in any of the groups.

**Conclusions:**

Nutritional support methods were not related to DGE after PPPD. EOF was feasible and safe after PPPD, and additional ENN should not be routinely administered to patients after PPPD.

**Clinical Trial Registration:**

ClinicalTrials.gov, identifier NCT03150615.

## Introduction

Pancreaticoduodenectomy (PD) is the standard procedure for patients with periampullary neoplasms. Although the mortality rate after PD has decreased to 1–3% at high-volume centers, morbidity rates remain very high, ranging from 30–50% (1, 2). Among the morbidities after PD, delayed gastric emptying (DGE) is one of the most common and troublesome postoperative complications, occurring in 20–40% of the patients (3–6). DGE manifests as excessive postoperative nausea, vomiting, and failure to progress with an oral diet (7). Although not lethal, DGE results in prolonged hospital stay, increased costs, and patient discomfort and could even negatively affect cancer-specific survival (8, 9). To date, the exact mechanism of DGE remains unclear, and conflicting conclusions have been reported in the literature.

Postoperative nutritional support is one of the main aspects of perioperative management and has been demonstrated to be relevant to postoperative outcomes (10). European Society for Parenteral and Enteral Nutrition (ESPEN) guidelines recommend the use of early enteral nutrition (EEN) in patients after gastrointestinal surgery for cancer, because compared to total parenteral nutrition, EEN has been demonstrated to be superior in strengthening the immune system, reducing complication rates, and maintaining gut integrity (11–14). However, with the development fast-track surgery and enhanced recovery program after surgery, early oral feeding (EOF) at will has been strongly recommended, while enteral tube feeding is recommended only on specific indications (15, 16).

However, most studies that support the use of EOF after PPPD are retrospective or nonrandomized trials, or compared EOF as a component of the Enhanced Recovery after Surgery (ERAS) protocol to EEN with standard perioperative care (17). A randomized trial comparing EOF versus early nasojejunal nutrition (ENN) in patients managed according to the ERAS protocol after PD has not been reported.

Previously, EEN was a popular practice in our center, and clinically relevant DGE was also a very prevalent complication, with an incidence rate of 35%. Since we introduced the EOF policy according to the ERAS guidelines in 2015, we noticed a significant decrease in the rate of clinically relevant DGE. Therefore, we carried out the present randomized clinical trial to validate whether EOF is superior to ENN in terms of postoperative outcomes, especially the rate of clinically relevant DGE, in patients undergoing PPPD.

## Patients And Methods

### Study Design

We conducted a prospective, single-center, three-arm, randomized controlled trial at the pancreas center of The First Affiliated Hospital of Nanjing Medical University between September 2016 and December 2017.

The hypothesis was that EOF would reduce the rate of clinically relevant DGE after PPPD compared with ENN.

### Ethical Approval, Safety, and Registration

The study protocol was approved by the ethical committee (2016-SR-121) and carried out according to the guidelines of the Good Clinical Practice and the Declaration of Helsinki. Written informed consent was obtained from all patients. The trial was registered in the Clinical Trials Register (NCT03150615).

### Participants

Patients aged >18 years scheduled for selective PPPD were eligible for the study. An additional inclusion criterion was an American Society of Anesthesiologists (ASA) score <4. Exclusion criteria were patients who refused to participate in the study, patients with a history of gastrointestinal surgery for any reason, patients undergoing types of Whipple procedure other than PPPD, and patients found to have unresectable disease during the operation.

### Randomization, Masking, and Blinding

Patients eligible for the study were randomly assigned in a 1:1:1 ratio to either the ENN, EOF, or saline group. Randomization was performed using a computer program with block sizes of 6 and 3. Sealed envelopes labeled with sequential study numbers were then prepared by a statistician before the study and opened by the statistician during the operation after confirmation that a PPPD was suitable for the disease. This was an open-label study.

### Interventions

On day 0, each patient underwent an open PPPD with Child reconstruction by the same team of surgeons. During the procedure, a nasojejunal nutrition tube (NJT) together with a nasogastric tube (NGT) was placed through the nasal cavity. The tip of the NJT was 20 cm distal to the duodenojejunostomy in the lumen of the jejunum, and the tip of the NGT was located in the lumen of the jejunum at the site of the choledochojejunostomy. The NGT was removed on the morning of postoperative day (POD) 3 in all three groups.

In the ENN group, patients were administered a standard enteral nutrition formula (Peptisorb, Nutricia Pharmaceutical [Wuxi] Co., China) via the NJT. Nutritional support was started on POD 2 at 30 ml/h, with an initial volume of 250 ml increasing gradually to normal intake in 72 h depending on the patient’s condition. In the saline group, equal amounts of saline were delivered via the NJT. In the EOF group, patients could drink water on POD 1, on POD 2, they were allowed a liquid diet, on POD 3, a semi-liquid diet, and on POD 4 and later, a solid diet was allowed without restrictions.

In all patients, additional parental nutrition was delivered from POD 1 until the recovery of full diet at 25 kcal/kg/day. The total calorie and protein intakes for the ENN group were aimed at 25–30 kcal/kg/day POD 5 and 1.2–1.5 g/kg/day, respectively. ENN and saline infusion were discontinued on POD 8. The NJT was removed on POD 8.

### Outcomes

The primary endpoint was the rate of clinically relevant DGE based on the International Study Group on Pancreatic Surgery (ISGPS) definition (7). Grade B DGE demands an NGT intubation or reinsertion between POD 8 and 14 or intolerance to a solid diet by POD 14; grade C demands an NGT intubation or reinsertion after POD 14 or intolerance to a solid diet by POD 21 (7).

Secondary endpoints were incidences of overall morbidity, postoperative pancreatic fistula, post-pancreatectomy hemorrhage, and abdominal infection, reoperation rate, length of hospital stay, readmission rate, and in-hospital mortality. Postoperative pancreatic fistula (POPF) and post-pancreatectomy hemorrhage (PPH) were also defined according to the ISGPS definitions and were considered only for grade B/C (18, 19). Chyle leak was defined as milky fluid drain effluent after POD 3. Abdominal infections were confirmed by microbiological analyses and positive cultures.

### Data Collection

After written informed consent was obtained from the patients to participate in the trial, demographic data, comorbidity, ASA score, body mass index (BMI), preoperative weight loss, and total albumin and prealbumin serum levels were collected.

Intraoperative data such as duration of surgery, estimated blood loss, need for blood transfusions, pancreatic texture (defined by the surgeon as soft, hard through palpation), and diameter of the main pancreatic duct were also recorded.

Details about complications and any additional treatment were recorded until the patients were discharged from the hospital or expired.

### Sample Size

Based on our previous experience, we estimated the incidence rate of clinically relevant DGE in the ENN group to be approximately 40%. The hypothesis was that with the adoption of the EOF protocol suggested by the ERAS protocol, the rate of clinically relevant DGE would be reduced from 40 to 10%. To achieve a power of 80% to detect differences in the two nutritional methods and with a two-sided test having a type I error of 0.05, it was calculated that 38 patients would be required in each group. A saline group was added to the study to neutralize the effect of the ENN group. That makes a total of 114 patients for the whole study. Data were analyzed according to intention-to-treat analysis.

### Statistical Methods

Statistical analysis was performed using Stata/MP 13.1 for Windows (StataCorp, Texas, USA). Descriptive data were reported as mean (standard deviation), median (interquartile), number of patients, and percentages. Continuous variables were analyzed using the analysis of variance (ANOVA) or Kruskal–Wallis test. Categorical variables were analyzed using the chi-square test or Fisher exact test, as appropriate. If the difference between groups was statistically significant, a *post hoc* analysis was performed using Fisher’s exact or chi-square pairwise comparison. Statistical significance was defined as p < 0.05.

## Results

### Participant Selection

**Figure 1:**
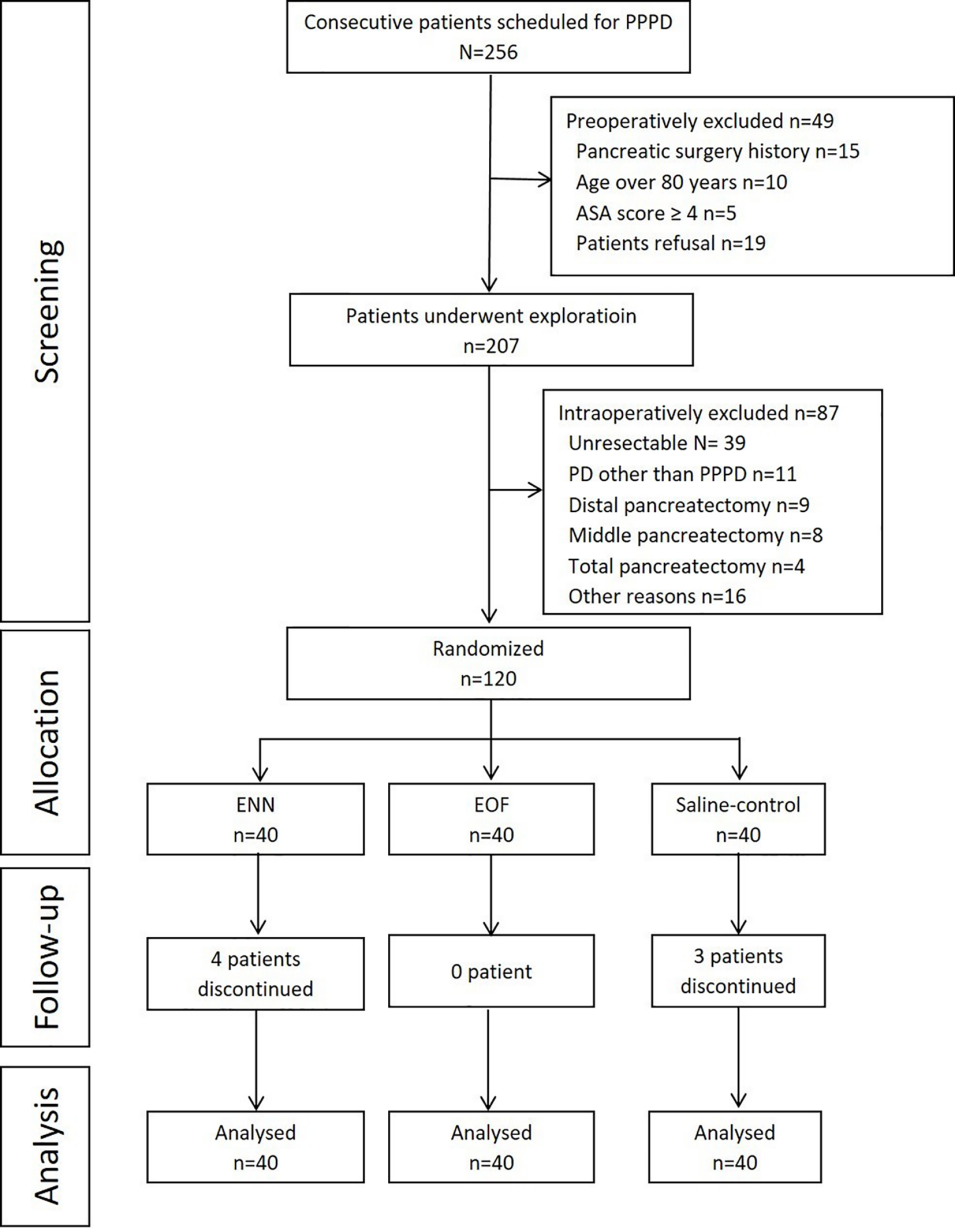
Flowchart of the study. PPPD, pylorus-preserving pancreaticoduodenectomy; PD, pancreaticoduodenectomy; ASA, American Society of Anesthesiologists; EOF, early oral feeding; ENN, early nasojejunal nutrition.

Among the 256 patients who were screened for eligibility, 49 were excluded preoperatively, and 87 patients were excluded intraoperatively (**Figure 1**). Finally, 120 patients were randomized and included in the analysis, with 40 patients allocated to the ENN group, 40 patients to the saline group, and 40 patients to the EOF group. In the ENN group, four patients did not finish the plan: three were due to dislodgement of the NJT, and one was due to occlusion of the NJT. In the saline group, dislodgement of the NJT occurred in three patients. All patients in the EOF group underwent treatment per protocol. No patients were lost to follow-up.

### Patient Baseline Data

Patient baseline characteristics are shown in **Table 1**. There were no significant differences among the treatment groups in terms of age, sex, BMI, indications, symptoms, preoperative biliary drainage, comorbidities, ASA score, preoperative serum albumin levels, and histological findings.

**Table 1:** Demographic and preoperative data.

	ENN n = 40	EOF n = 40	Saline n = 40	p
Age (years)	63.4 (9.2)	60.4 (9.8)	60.2 (9.8)	0.253
Sex ratio (M/F)	29/11	23/17	25/15	0.362
Diabetes (n, %)	11 (27.5%)	13 (32.5%)	7 (17.5%)	0.296
Hypertension	17 (42.5%)	16 (40.0%)	13 (32.5%)	0.632
Drinking history	11 (27.5%)	8 (20.0%)	9 (22.5%)	0.722
Smoking history	11 (27.5%)	10 (25.0%)	8 (20.0%)	0.727
Weight loss	14 (35.0%)	11 (27.5%)	8 (20.0%)	0.324
Preoperative BMI (mean ± SD) (kg/m^2^)	22.3 ± 3.4	23.1 ± 3.4	23.6 ± 3.6	0.211
ASA score				0.298
I	3 (7.3%)	1 (2.5%)	5 (12.5%)	
II	24 (58.5%)	30 (75.0%)	24 (60.0%)	
III	14 (34.2%)	9 (22.5%)	11 (27.5%)	
Total protein (mean ± SD) (g/L)	64.2 ± 6.3	63.4 ± 7.8	63.2 ± 4.7	0.793
Preoperative albumin (mean ± SD) (g/L)	38.7 ± 4.1	39.4 ± 4.7	39.0 ± 5.0	0.789
Pathological diagnosis (n, %)				0.765
PDAC	20 (50.0%)	18 (45.0%)	17 (42.5%)	
Duodenal cancer	6 (15.0%)	5 (12.5%)	8 (20.0%)	
Distal bile duct cancer	4 (10.0%)	3 (7.5%)	6 (15.0%)	
Others	10 (25.0%)	14 (35.0%)	9 (22.5%)	

ENN, early nasojejunal nutrition; EOF, early oral feeding; BMI, body mass index; SD, standard deviation; PDAC, pancreatic ductal adenocarcinoma.

The treatment groups were also comparable in terms of operation time, estimated intraoperative blood loss, and intraoperative transfusion requirement (**Table 2**).

**Table 2:** Intraoperative characteristics.

	ENN	EOF	Saline	p
Operation time (minutes, mean, [SD])	218 (62)	205 (61)	214 (61)	0.643
Estimated blood loss (mL, median, [IQ])	200 (100, 300)	200 (100, 300)	150 (100, 400)	0.788
Need of blood transfusion (n, %)	2 (4.9%)	1 (2.5%)	3 (7.5%)	0.591
Pancreatic texture				0.202
Soft	6	12	12	
Hard	34	28	28	
Main pancreatic duct				0.122
<3.0 mm	21	12	16	
≥3.0 mm	19	28	24	

ENN, early nasojejunal nutrition; EOF, early oral feeding; SD, standard deviation; IQ, interquartile range.

### Primary Endpoint Analysis

Overall, the rate of clinically relevant DGE was 17.5, 10.0, and 32.5% in the ENN, EOF, and saline groups, respectively. The overall variability between the groups was statistically significant (p = 0.038). Stepwise comparison showed that the saline-control group had a higher rate of DGE compared to that in the EOF group (p = 0.014), while there were no significant differences between the ENN group and the other two groups (**Table 3**).

**Table 3:** Postoperative outcomes.

	ENN	EOF	Saline	p	Stepwise comparison		
	(A)	(B)	(C)		A vs. B	A vs. C	B vs. C
DGE grade B/C	7 (17.5%)	4 (10.0%)	13 (32.5%)	**0.038**	0.330	0.121	**0.014**
B	2 (5.0%)	3 (7.5%)	7 (17.5%)				
C	5 (12.5%)	1 (2.5%)	6 (15.0%)				
Overall morbidity	12 (30.0%)	9 (22.5%)	21 (52.5%)	**0.014**	0.446	**0.041**	**0.006**
POPF	6 (15.0%)	7 (17.5%)	11 (27.5%)	0.335			
PPH	1 (2.5%)	1 (2.5%)	2 (5.0%)	1.000			
Chylous fistula	2 (5.0%)	1 (2.5%)	0 (0)	0.544			
Digestive fistula	1 (2.5%)	0 (0)	0 (0)	1.000			
Incision dehiscence	1 (2.5%)	0 (0)	0 (0)	1.000			
Abdominal infection	3 (7.5%)	2 (5.0%)	5 (12.5%)	0.466			
Reoperation	0 (0)	0 (0)	0 (0)	1.000			
Readmission	1 (2.5%)	2 (5.0%)	3 (7.5%)	0.591			
LOS	14 (11, 20)	13 (11, 17)	15 (11, 20)	0.378			
Mortality	0 (0)	0 (0)	0 (0)	1.000			

ENN, early nasojejunal nutrition; EOF, early oral feeding; DGE, delayed gastric emptying; POPF, postoperative pancreatic fistula; PPH, postpancreatectomy hemorrhage; LOS, length of postoperative stay. Bolded values mean a p < 0.05 with the chi-square test.

### Secondary Endpoint Analysis

Overall morbidity was significantly different among the three groups. Stepwise comparisons showed that the saline group had a higher rate of morbidity compared to the ENN (52.5% vs. 30.0%, p = 0.041) and EOF groups (52.5% vs. 22.5%, p = 0.006), while there was no significant difference between the ENN and EOF groups (30.0% vs. 22.5%, p = 0.446).

The rates of clinically relevant POPF in the saline group were higher than compared to the ENN and EOF groups (27.5, 15.0, and 17.5%, respectively), but these differences were not statistically significant (p = 0.335). There was also no significant difference in PPH (ENN 2.5%, EOF 2.5%, saline 5.0%; p = 1.000), chylous fistula (ENN 5.0%, EOF 2.5%, saline 0%; p = 0.544), or abdominal infection (ENN 7.3%, EOF 5.0%, saline 12.5%; p = 0.466) between the groups. Digestive fistula occurred in one patient in the ENN group, and incision dehiscence occurred in one patient in the ENN group. All complications were managed conservatively or with the intervention therapy. There was no reoperation or mortality in any of the three groups.

## Discussion

This study demonstrates that ENN does not have an advantage over EOF in terms of clinically relevant DGE rates. Moreover, ENN was associated with a slightly higher rate of clinically relevant DGE and overall morbidity than EOF. These results indicate that additional ENN after PPPD is not warranted, and the EOF strategy could be a safe and acceptable technique after PPPD. Our study provides new evidence to support EOF after PPPD.

DGE is a very common complication of PD. Although tremendous efforts have been made to investigate DGE, and many factors, such as inflammation, postoperative hyperglycemia, ischemia, gastric atony, motilin levels, and type of surgical procedure, have been proposed to be related to DGE, the exact pathogenesis of DGE still remains unclear, and the prevention and treatment strategy for DGE has not been established yet. Nutritional support methods are associated with postoperative gastric function and postoperative recovery (20). Previously, EEN was believed to be safe and well tolerated in patients after PD (12, 21), and enteral nutrition could reduce DGE after PD (22). EEN is recommended by international guidelines and is a widely adopted routine feeding strategy after PD (14, 23, 24).

However, this concept has been challenged recently. During the study period, Perinel et al. reported that nasojejunal EEN increased the overall postoperative complication rate compared with total parenteral nutrition, and should not be recommended in terms of safety and feasibility (25). In addition, a meta-analysis showed that there is no evidence to support either routine enteral or parenteral feeding after PD, and further suggested that an oral diet may be considered as the preferred routine feeding strategy after PD (26). Several other studies suggested that the best nutritional method after PD is a normal oral diet after surgery, as recommended by the ERAS protocol (27–29). More recently, this recommendation of early resumption of oral intake was endorsed by the ISGPS in 2018 (30).

However, in most studies, EOF was just one of the multimodal strategies of ERAS, and the actual impact of EOF was investigated in one study with similar postoperative management, except for the feeding strategy (17). In their observational, nonrandomized, prospective cohort study with historical controls, Gerritsen et al. found that an EOF strategy after PD reduced the time to resumption of adequate oral intake and length of hospital stay without negatively affecting postoperative morbidity, including clinically relevant DGE (17). Our results were comparable to the findings of Gerritsen et al. in terms of the incidence of clinically relevant DGE; however, we did not find a reduction in the length of hospital stay.

A special innovation point of this trial was that we introduced a saline group, which, to the best of our knowledge, has never been done in the literature. This saline group had the highest rate of clinically relevant DGE and most morbidities in the three groups. When compared to the ENN group, the saline group ingested lower calories, which might indicate that jejunal distension did not play a role in the development of DGE. Energy support was not the only reason for DGE, while the presence of intestinal contents may be the reason for this common complication after PPPD. This may be mediated by stress-dependent mechanoreceptors. In a rat experiment, Dr. Bárdos found that distension decreased fluid intake in an intensity-dependent manner and suggested that mild discomfort is a physiological satiety factor (31). Moreover, the saline group was not only associated with a significantly higher incidence of DGE than the EOF group but was also associated with more overall morbidities than both the ENN and EOF groups. However, no significant differences were identified between the ENN and EOF groups. Therefore, in patients with PPPD, the use of enteral nutritional therapy is more important than the administration methods of enteral nutritional therapy.

This study has several limitations. First, this was a relatively small, single-center study with inherent bias. Furthermore, this study focused only on PPPD, and all operations were performed by a single team of surgeons. This optimized the uniformity of the baseline data. However, this also makes the results of the present study less generalizable. Second, the initial power calculations were based on our previous experience that the ENN group would have a 40% clinical DGE rate. However, the results showed that the ENN had less than half of the assumed rate. There is a risk that the results presented here are related to a type II error. Third, patients were not randomized according to their nutritional state, and no blinding was suitable for this study because of its nature.

## Conclusions

This randomized study demonstrated that, compared to saline control, EEN resulted in lower DGE and overall morbidity. However, in terms of nutritional support methods, ENN was not superior to EOF in terms of clinically relevant DGE rates and overall morbidities. Additional ENN support should not be routinely administered to patients after PPPD.

## Data Availability Statement

The raw data supporting the conclusions of this article will be made available by the corresponding authors on reasonable request.

## Ethics Statement

The studies involving human participants were reviewed and approved by the The First Affiliated Hospital of Nanjing Medical University. The patients/participants provided their written informed consent to participate in this study.

## Author Contributions

YM and JiW conceived the study and are the principal investigators. XL, QC, YF, ZL, JC, FG, QL, JuW, WG, KJ, and CD contributed to the design and oversaw the study conduct. XL, QC, and YF wrote the manuscript. XL was responsible for the statistical analyses. JuW, ZL, and WG contributed to the quality control. All authors contributed to the article and approved the submitted version.

## Funding

This study was supported by the Project of Invigorating Health Care through Science, Technology and Education, Jiangsu Provincial Medical Outstanding Talent (JCRCA2016009).

## Conflict of Interest

The authors declare that the research was conducted in the absence of any commercial or financial relationships that could be construed as a potential conflict of interest.
